# Case report: rTMS in combination with aripiprazole and sodium valproate for the maintenance treatment of rapid cycling bipolar disorder

**DOI:** 10.3389/fpsyt.2023.1070046

**Published:** 2023-03-16

**Authors:** Siyi Tao, Bing Chen, Xiaofang Xu, Shaohua Hu, Jing Lu

**Affiliations:** ^1^Department of Psychiatry, The First Affiliated Hospital, Zhejiang University School of Medicine, Hangzhou, China; ^2^Department of Nursing, The First Affiliated Hospital, Zhejiang University School of Medicine, Hangzhou, China; ^3^The Key Laboratory of Mental Disorder Management in Zhejiang Province, Hangzhou, China

**Keywords:** repetitive transcranial magnetic stimulation, bipolar disorder, rapid cycling, aripiprazole, sodium valproate

## Abstract

As a safe neuromodulation therapy, rTMS is applied to treat a variety of psychiatric and neurological disorders. Additionally, both aripiprazole and sodium valproate are effective in the treatment of rapid cycling bipolar disorder. This case reports a female patient with a 17-year history of bipolar disorder who developed rapid-circulation bipolar disorder 5 years prior to presentation. After combined treatment with rTMS, aripiprazole, and sodium valproate, the patient’s mood remained stable and she was able to live and work normally.

## 1. Background

Bipolar disorder (BD) is a disabling and severe mental illness characterized by an early onset, frequent recurrence, a chronic course, and high risk of comorbidities and suicide (1). People diagnosed with BD often display behaviors such as self-mutilation and harming others, which affect their quality of daily life and health (2). At present, BD is mainly treated by using pharmacotherapy, mainly using the combination of mood stabilizers and antipsychotic drugs (3). Rapid cycling, the most malignant type of BD and one of the specifiers used for BD in the Diagnostic and Statistical Manual of Mental Disorders, shows a prevalence of 25–43% in patients with BD; its annual prevalence ranges from 5 to 33.3%, while lifetime prevalence ranges from 25.8–43% (4). It is also closely associated with more serious disease, longer disease duration, and higher risk of suicide (5, 6). However, its etiology is unclear and its therapies are very complex without a consensus, additionally, most patients with rapid cycling BD are resistant to treatment (7).

Repetitive transcranial magnetic stimulation (rTMS), a safe and non-invasive neuromodulation therapy, is applied in a variety of psychiatric and neurological disorders (8–10), and several case reports suggested that it also plays an important role in the treatment of BD (11, 12). In addition, aripiprazole and sodium valproate alone have been shown to be effective in the treatment of rapid cycling BD (13, 14). Therefore, in this case, we combined rTMS with the two drugs to exert synergistic effects.

We report a case of a woman with a 17-year history BD, continuing to present with rapid cycling BD 5 years before admission. After treatment with rTMS combined with aripiprazole and sodium valproate, the patient showed significant improvement in rapid cycling.

## 2. Case presentation

**Figure 1:**
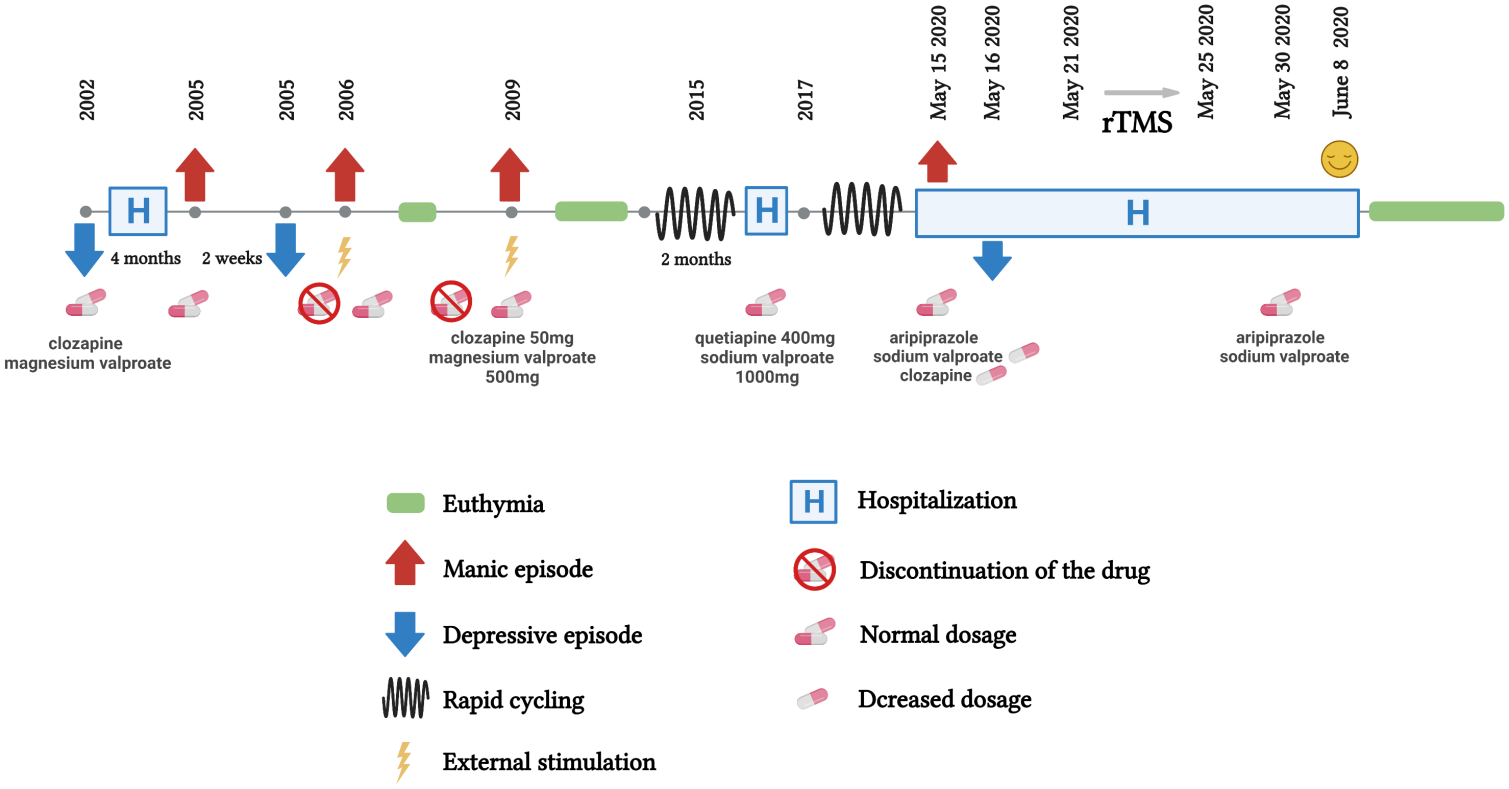
Symptoms and treatment course of the patient.

The patient, a 38-year-old woman with rapid cycling bipolar disorder, was hospitalized with “repeated transitions between depressive and manic episodes.” In 2002, without obvious cause, the patient suffered from low mood, was less talkative, experienced poor sleep, was diagnosed with “depression” for the first time, and was treated with “clozapine, magnesium valproate” (specific dose unknown), taking medication regularly after discharge. In 2005, the patient developed high mood without any incentive, was talking a lot and speaking fast, and had highly active thoughts, which lasted about half a month. The patient gradually appeared depressed, showing increases in sleep duration. In 2006, after being stimulated, the patient showed high mood again and was stabilized after medication. In 2009, high mood, excitement, talkativeness, high energy, and a decreased sleep demand occurred again, which lasted for about half a month, and were stabilized after administration of clozapine (50 mg) and magnesium valproate (500 mg). Depressive and manic episodes alternated every 2 weeks in 2015 and, after the patient received “sodium valproate 1,000 mg, quetiapine 400 mg,” she still experienced moderate transitions between manic and depressive episodes, lasting for about one and a half years. The patient had biphasic conversion every half month again in 2017, and could not work normally. The patient’s complete course is shown in Figure 1.

On the day of admission to our institute (May 15), the patient was in a happy mood, willing to communicate with others, was diagnosed with BD (rapid cycling), and was currently in a hypomanic episode, the physical examination revealed normal blood pressure, pulse, respiratory rate, electrocardiogram and other physical signs, the chest computed tomographic and brain MRI also showed no obvious abnormal signs. Aripiprazole (2.5 mg/d) and sodium valproate (1,000 mg/d) were used as mood stabilizers while clozapine (50 mg/d) was gradually reduced. However, on the second day of admission (May 16), the patient transitioned into a depressive state, with low mood, and unwillingness to speak and to be active, and poor appetite. From May 16 to May 22, the patient showed depressed emotions, a lack of energy, poor sleep and appetite, normal logic, and no negative ideas and positive signs in neurological examinations. With a clear diagnosis of rapid cycling BD, the previously used clozapine was not our first treatment recommendation, and the dose was reduced to 25 mg/d while the dose of aripiprazole was gradually increased to 15 mg/d, combined with sodium valproate (1,000 mg/d) to stabilize mood and alprazolam (0.2 mg/d) to improve sleep. rTMS (1 Hz, 47% of motor threshold, 600 stimuli/d) was used continuously from May 21 to 25 as a treatment.

**Figure 2:**
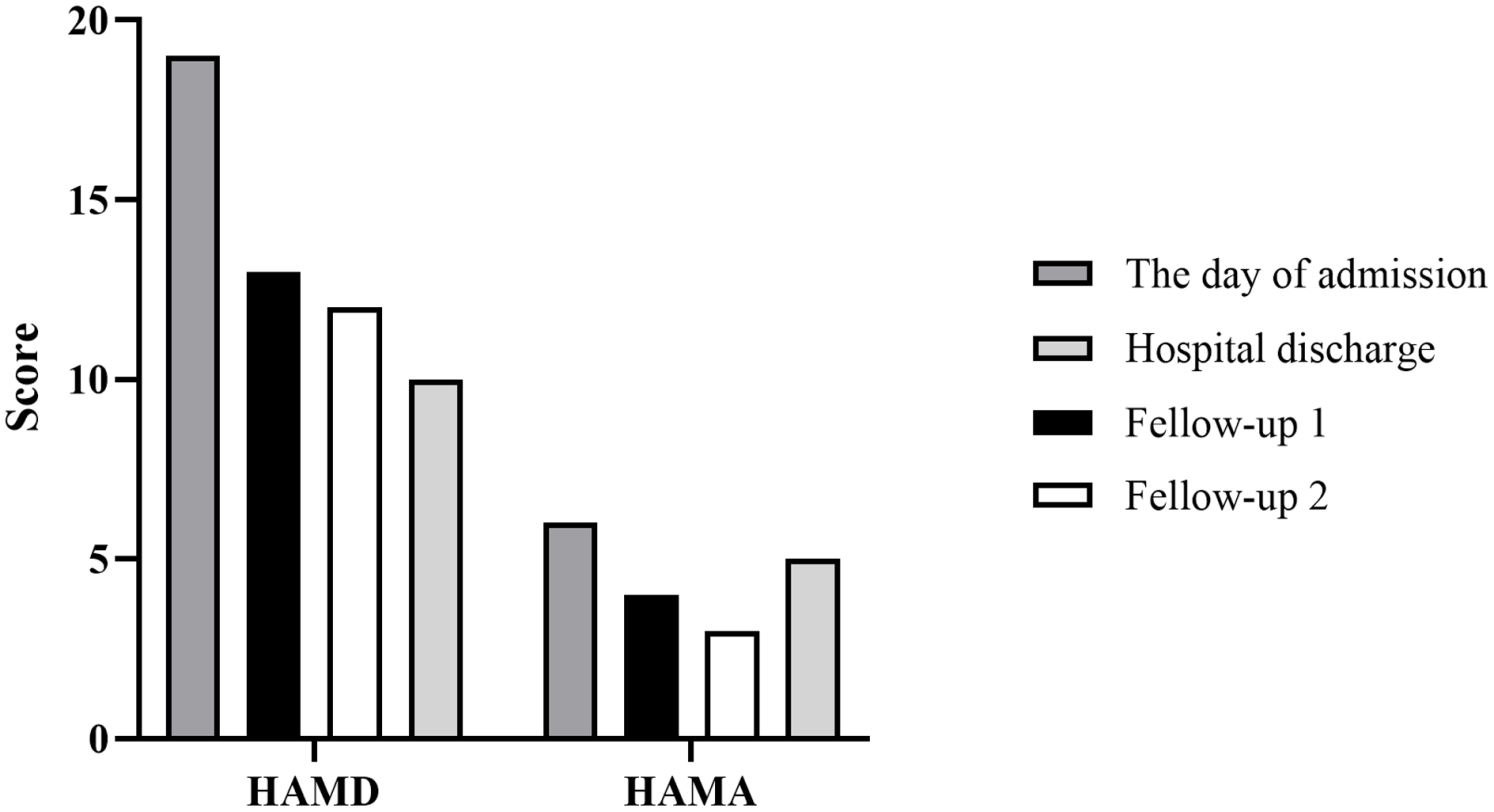
Histogram depicting score changes on HAMD and HAMA at four time points. The day of admission: May 15, 2020; Hospital discharge: June 8, 2020; Fellow-up 1 and 2: June 23, 2020 and August 15, 2020, respectively.

On May 26, the patient’s mood was stable; meanwhile, her fatigue improved, and she was willing to get up and move, smiled when communicating, and spoke louder than before. On May 30, the dose of aripiprazole was increased to the recommended dose (20 mg/d) while clozapine was withdrawn. The patient remained emotionally stable, and her self-knowledge was complete. On June 8, with the improvement of sleep and Hamilton Depression Scale (HAMD) and Hamilton Anxiety Scale (HAMA) scores (as shown in Figure 2), the patient was discharged with prescriptions for aripiprazole and sodium valproate.

**Figure 3:**
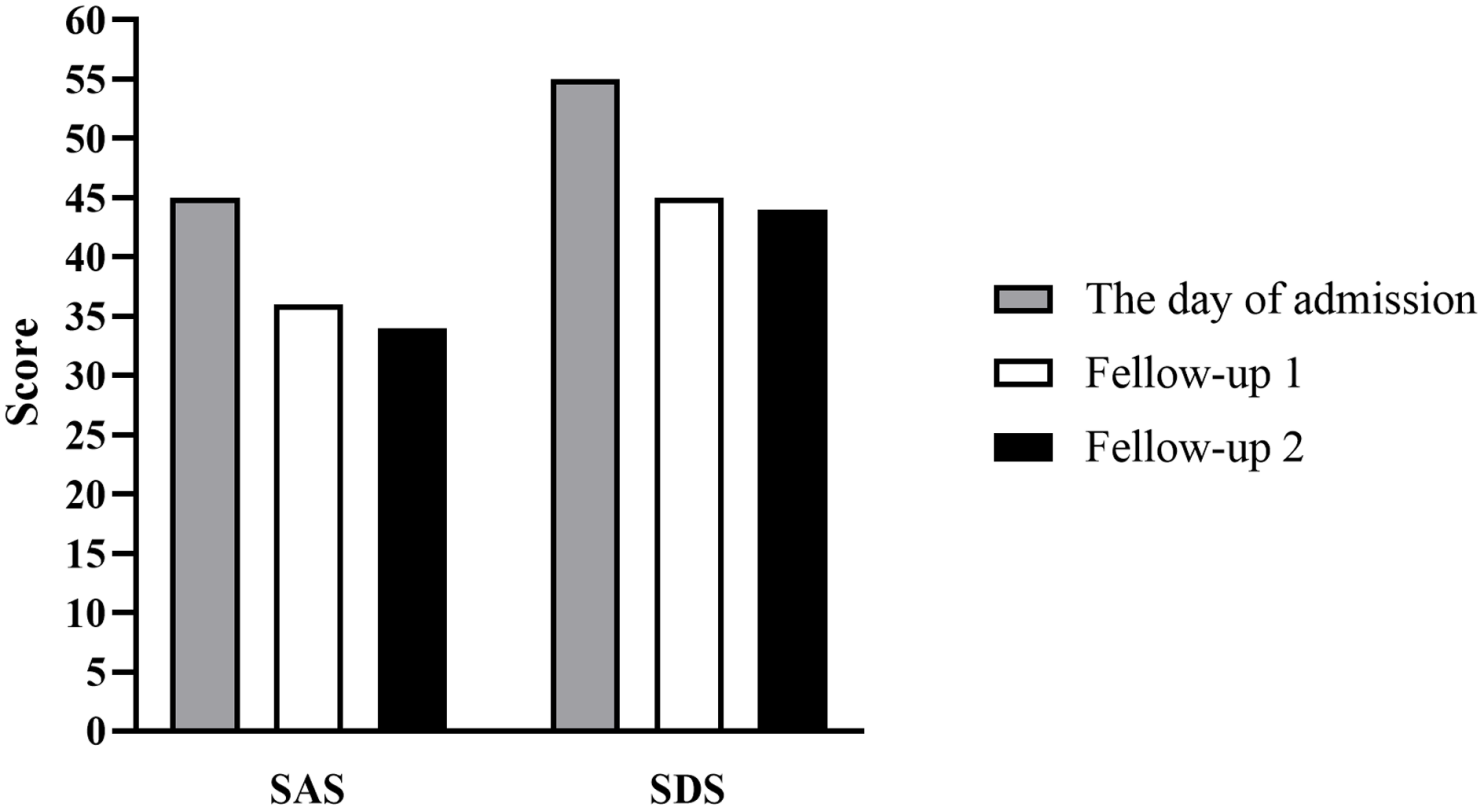
Histogram depicting score changes on SAS and SDS at three time points. The day of admission: May 15, 2020; Fellow-up 1 and 2: June 23, 2020 and August 15, 2020, respectively.

**Figure 4:**
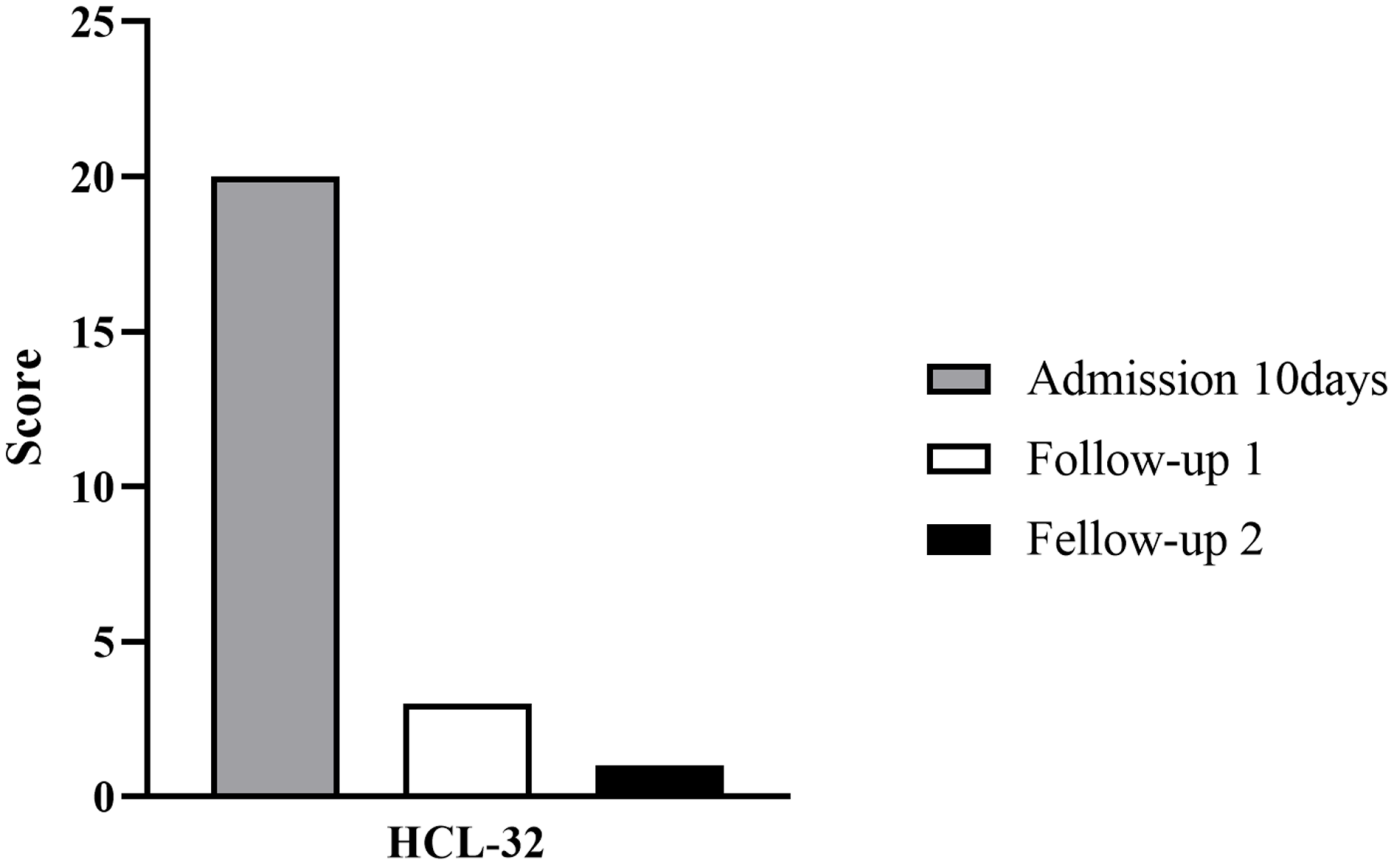
Histogram depicting score changes on HCL-32 at three time points. HCL-32: 32-item hypomania checklist. Admission 10 days: May 26, 2020; Fellow-up 1 and 2: June 23, 2020 and August 15, 2020, respectively.

With the continuous combined administration of aripiprazole and sodium valproate after being discharged, the patient’s depression was significantly improved, resulting in significant scores of the Self-rating Depression Scale (SDS), Self-rating Anxiety Scale (SAS), HAMD and HAMA at two follow-up visits (Figures 2, 3). And as shown in 32-item hypomania checklist (HCL-32), the patient’s manic symptoms were also relieved after 40 days of treatment and remained stable at subsequent follow-ups (Figure 4).

## 3. Discussion

Most patients with BD in the acute phase of rapid cycling are women under the age of 40 (15). However, the etiology of rapid cycling BD remains unclear, although a causal or triggering role of antidepressants and hypothyroidism is implicated. If the women are younger than 30 years old when experiencing their first BD episode, they are more likely to develop rapid cycling BD. The course of the patient described here, showed transitions between depressive and manic states many times over 20 years. In the 5 years prior to admission to our hospital, the patient showed rapid cycling BD characterized by obvious and regular mood episodes, with a cycle duration of depressive and manic episodes of roughly 2 weeks.

The combination of mood stabilizers and antipsychotics is the main treatment for BD. Lithium was the first mood stabilizer, and risperidone was the first antipsychotic drug suggested in the Guidelines for the Prevention and Treatment of Bipolar Disorder (16). However, treatments and patient situations vary in different regions (17, 18). Sodium valproate combined with aripiprazole have shown significant effects in the treatment of BD manic episodes, which can effectively improve the quality of life and sleep quality of patients with mild adverse reactions and high safety (19). Currently, aripiprazole is the only dopamine system stabilizer that keeps serotonin and dopamine levels in the brain in a stable balance, which can improve positive symptoms and depression in patients with BD. This can also improve patients’ cognitive function (20). Aripiprazole is more suitable for female patients with manic episodes of BD because its side effects, such as menstrual changes, lactation, drowsiness, increased appetite, and weight gain, are significantly less severe than those of quetiapine, and its improvement in the quality of life is better than quetiapine in female patients (21).

Although aripiprazole is associated with lower risk of side effects such as weight gain, dyslipidemia and diabetes compared with other antipsychotics, it can still induce side effects such as akathisia and compulsive behavior (22–24). In addition, valproate has been found to induce acute pancreatitis in BD patients (25). For women of childbearing age with BD, the use of valproate during pregnancy is problematic due to its teratogenic effects and potential neurodevelopmental damage to the fetus (26, 27). A long-term combination of aripiprazole and valproate has been found to induce acute pancreatitis in BD patients with an increased risk of extrapyramidal (28). Previous studies have shown that rTMS in patients with BD seemed to improve cognitive domains in euthymic patients, while its effects during acute phases, especially depressive phases, appeared limited (8). However, the present case showed that, after continuous treatment with rTMS in a depressive period of rapid cycling BD, there was a significant improvement in symptoms, suggesting that rTMS may also play an important role in the treatment of BD during depressive episodes or that rTMS combined with aripiprazole and sodium valproate may play a synergistic role in the treatment of rapid cycling BD.

In summary, the combined treatment with rTMS, aripiprazole, and sodium valproate showed good effects, which demonstrated the clinical value of the combined use of these treatments for rapid cycling BD.

## 4. Strengths and limitations

A limitation of the present work is that it was based on the description of a single patient. However, this is a case of successful treatment of rapid cycling BD, indicating that rTMS may play a crucial role, which may provide a reference for future applications of rTMS in the treatment of BD. Additionally, aripiprazole in combination with sodium valproate is an effective and safe treatment for rapid cycling BD.

## Data availability statement

The original contributions presented in the study are included in the article/supplementary material, further inquiries can be directed to the corresponding authors.

## Ethics statement

Written informed consent was obtained from the individual(s) for the publication of any potentially identifiable images or data included in this article.

## Author contributions

ST and SH interviewed and treated the patient who is the subject of this case report, managed the literature searches, and wrote the first draft of the manuscript. BC, XX, and JL rewrote portions of the first draft of the manuscript and made revisions to the manuscript. All authors contributed to the article and approved the submitted version.

## Funding

We sincerely thank the support of funds from the Medical Science and Technology Project of Zhejiang Province (2022RC024), the National Natural Science Foundation of China (82271561).

## Conflict of interest

The authors declare that the research was conducted in the absence of any commercial or financial relationships that could be construed as a potential conflict of interest.

## Publisher’s note

All claims expressed in this article are solely those of the authors and do not necessarily represent those of their affiliated organizations, or those of the publisher, the editors and the reviewers. Any product that may be evaluated in this article, or claim that may be made by its manufacturer, is not guaranteed or endorsed by the publisher.
